# Assessing the Future of Solid Tumor Immunotherapy

**DOI:** 10.3390/biomedicines10030655

**Published:** 2022-03-11

**Authors:** Prajna Guha, Kara R. Heatherton, Kyle P. O’Connell, Ian S. Alexander, Steven C. Katz

**Affiliations:** 1 Roger Williams Medical Center, Immuno-Oncology Institute, Providence, RI 02908, USA; kr.heatherton@gmail.com (K.R.H.); kyle.oconnell4794@gmail.com (K.P.O.); ianalexander2023@u.northwestern.edu (I.S.A.); steven.katz@trisaluslifesci.com (S.C.K.); 2 Department of Surgery, Boston University Medical Center, Boston, MA 02118, USA; 3 TriSalus™ Life Sciences, Inc., Westminster, CO 80031, USA; 4 McCormick School of Engineering, Northwestern University, Evanston, IL 60208, USA; 5 Department of Medicine, Roger Williams Medical Center, Providence, RI 02908, USA

**Keywords:** adoptive cell therapy, CAR-NK, CAR-T, cytokine release syndrome, immune checkpoint inhibitors, myeloid derived suppressor cells, On-target-off-tumor-toxicity, single chain variable fragment (scFv), tumor microenvironment

## Abstract

With the advent of cancer immunotherapy, there has been a major improvement in patient’s quality of life and survival. The growth of cancer immunotherapy has dramatically changed our understanding of the basics of cancer biology and has altered the standards of care (surgery, radiotherapy, and chemotherapy) for patients. Cancer immunotherapy has generated significant excitement with the success of chimeric antigen receptor (CAR) T cell therapy in particular. Clinical results using CAR-T for hematological malignancies have led to the approval of four CD19-targeted and one B-cell maturation antigen (BCMA)-targeted cell therapy products by the US Food and Drug Administration (FDA). Also, immune checkpoint inhibitors such as antibodies against Programmed Cell Death-1 (PD-1), Programmed Cell Death Ligand-1 (PD-L1), and Cytotoxic T-Lymphocyte-Associated Antigen 4 (CTLA-4) have shown promising therapeutic outcomes and long-lasting clinical effect in several tumor types and patients who are refractory to other treatments. Despite these promising results, the success of cancer immunotherapy in solid tumors has been limited due to several barriers, which include immunosuppressive tumor microenvironment (TME), inefficient trafficking, and heterogeneity of tumor antigens. This is further compounded by the high intra-tumoral pressure of solid tumors, which presents an additional challenge to successfully delivering treatments to solid tumors. In this review, we will outline and propose specific approaches that may overcome these immunological and physical barriers to improve the outcomes in solid tumor patients receiving immunotherapies.

## 1. Introduction

Cancer immunotherapy dates back to William B. Coley in 1891 when he began treating cancer patients with different combinations of bacteria and their derivatives to provoke an immune response [1]. While the immune system was not understood or recognized at that time, Coley and others observed that patient responses to infection were on occasion associated with tumor regression. Although his work with ”Coley’s Toxins” was not widely accepted in his time, he continued to treat over 1000 patients during his 40 years as a physician and is now regarded as the “Father of Cancer Immunotherapy” [2].

Cancer immunotherapy is transforming multidisciplinary cancer care and opening new therapeutic avenues. Current types of immunotherapy that have been explored include monoclonal antibodies (mAb), immune checkpoint inhibitors (ICI), oncolytic viral platforms (OV), cancer vaccines, adoptive cell therapy (ACT), and various combinatorial approaches [3]. In this review article, we will focus on the current standards of care in cancer immunotherapy, with a strong focus on the promise and limitations of ACT [4], which includes tumor-infiltrating lymphocytes (TIL’s), CAR-T cells, and CAR-natural killer cells (CAR-NK).

## 2. Standard of Care Therapies

When a patient is first diagnosed with cancer, various treatments can be offered. The appropriate management plan for a given patient depends on several factors including disease type, sites of disease, and patient physiologic status. For years, the standard cancer treatments included surgery, chemotherapy, radiation, or a combination approach. Surgical resection is potentially curative in selected cases, but most advanced solid tumor patients are not candidates for this approach [5]. Most patients with advanced solid tumors require multidisciplinary care including chemotherapy or radiation [6].

A major limitation of cytotoxic chemotherapy drugs is that the drugs generally lack specificity and attack both normal and tumor cells, causing potentially severe side effects [7]. Radiation therapy is often used in combination with chemotherapy or surgery because the use of radiation alone cannot cure most forms of cancer [8]. Typical adverse effects include dry, itchy, and swelling skin, and overall stiffness and fatigue [9]. Even when combinatorial treatment based on surgery, chemotherapy, and radiation achieve disease control, durability or cure are quite rare. Cancer immunotherapy is becoming increasingly embedded in multidisciplinary cancer care in part due to its ability to provide durable disease control in a higher proportion of patients under some circumstances. Expanding the impact of immunotherapy to include more solid tumor types will require further advances that overcome critical barriers related to immunosuppression and targeted delivery, among others.

## 3. Basics of Immunotherapy

The immune system is known to play a pivotal role in tumorigenesis, hence the role of immunotherapies in treating different kinds of tumors has become crucial. In the 1990s, the first tumor associated antigen was cloned (melanoma associated antigen 1) and immunogenic tumor antigens were discovered, implying that the immune system can recognize and clear them [10]. Interferon α2 was approved by the US FDA for adjuvant treatment of stage IIb/III melanoma in 1995 and IL-2 was FDA approved for the treatment of metastatic melanoma and renal cell carcinoma [10]. In the 21st century, several ICIs were evaluated and approved for the immunotherapy of different types of cancers.

There have been many immunotherapy clinical trials with varying levels of success. Most of these trials have involved the use of mAbs, checkpoint inhibitors, OVs, cancer vaccines, and ACT [11,12]. Thus far, immunotherapies combating hematological cancers and a select group of solid tumors have demonstrated success [13]. New cases of leukemia, lymphoma, and myeloma (hematological cancers) accounted for 9.9% of new cancer cases diagnosed in the US in 2020, while solid tumors made up the rest of the 90% of cancer cases [14]. Immunotherapies, such as CAR-T cells for treating hematological cancers, are typically administered intravenously. Once in circulation, CAR-T cells have easier access to target cells in the setting of hematologic malignancies as compared to solid tumors. Although there has been success in hematological cancers, especially targeting CD19, most of the clinical trials focused on solid tumors have yielded less encouraging results [15]. There are several obstacles within solid tumor TMEs, such as regulatory T cells (Tregs), myeloid derived suppressor cells (MDSCs), tumor-associated macrophages (TAMs), suppressive cytokines (IL-10, TGF-β), hypoxia, and barriers to effective drug delivery, including high intra-tumoral pressure. Systemic infusion of immunotherapies can cause on-target-off-tumor toxicities (OTOTT) and poor trafficking of the CAR-T cells to the tumor site further compromises the therapeutic index [16,17]. Success with solid tumor cell therapy will require a greater understanding of the TME, innovative cell engineering strategies, and appropriate delivery techniques.

## 4. Solid Tumor Immunotherapy Barrier Overview

Clinical success with immunotherapy for solid tumors has, in many cases, been very difficult to achieve. While surgical resection, chemotherapy, and radiation are often the primary treatment modalities for solid tumors, these methods may fail initially or be unable to provide durable disease control. To achieve greater and more consistent success in patients with advanced solid tumors, immunotherapy must be enabled to overcome challenges posed by solid tumors that are not present in hematological cancers.

### 4.1. Tumor Microenvironment (TME) General Features and Soluble Mediators

The vasculature in solid tumors is highly abnormal, consisting of capillaries that are often leaky with markedly impaired perfusion [18,19]. This irregular stroma and vasculature typically promote hypoxic and acidic conditions, creating a challenging environment for cell therapy performance [20]. The typical solid tumor TME also limits the penetration of cell therapy products due to high intra-tumoral pressure, leaving much of the tumor mass inaccessible and hence it remains untreated [21,22]. The solid tumor TME also presents immunologic challenges due to immunosuppressive cytokines and suppressor cells, the nature of which may vary by anatomic site and disease histology [23,24]. Immunosuppressive programming within solid tumor TMEs often represents a broad network of cells and soluble mediators.

MDSCs and Tregs are major components that contribute to the immunosuppressive TME in solid tumors. MDSCs and Tregs are known to expand in several murine tumor models and promote T cell dysfunction [25]. Furthermore, the immunologic landscape and TME differ between different organs and should be accounted for when targeting these cell types based on the organ of interest. For example, the liver, an inherently immunosuppressive organ, was found to promote a unique suppressive program among MDSC compared with the lung in models of metastatic disease [26]. The implications of organ-specific immunosuppressive programming may be clinically relevant, as different disease sites within the same patients could require tailored approaches.

Other contributing factors to a suppressive solid tumor TME include chemokines and cytokines produced by immune cells that activate transcription factors such as AP-1, NFκB, and STAT3, which support malignant cell proliferation and survival [27]. NFκB and STAT3 are highly activated in many types of cancer and control cell survival, proliferation, and growth, as well as angiogenesis, invasiveness, and chemokine and cytokine production [28]. Cytokines regulated by NF-κB and STAT3 can either be tumor-inducing (TNF, IL-23, IL-6) or tumor-inhibiting (IFNα, IFNγ, TRAIL) [28]. In colitis associated colorectal cancer and hepatocellular carcinoma, tumor promotion is supported by IL-6 in a STAT3-dependent signaling mechanism [29]. Critical growth factors and cytokines, including IL-6, IL-11, IL-22, HGF, and EGF, in addition to oncogenic tyrosine kinases such as c-Met and Src, cause STAT3 dependent activation of tumor growth [29,30,31,32]. Not only do NFκB and STAT3 directly drive tumor cell biology, but they have also been implicated in the programming of suppressive immune cells, which may in turn drive failure of both endogenous immunity and immunotherapeutics [4,33,34].

TNF-α is a known critical player in tumor signaling pathways and immune cell manipulation within the TME and is mainly produced by activated macrophages, T lymphocytes, and natural killer (NK) cells. In cancer immunotherapy, TNF-α acts as a mediator of anti-tumor immune responses and several immunotherapies have shown reduced anti-tumor activity in the presence of TNF-α antagonists [35,36]. TNF-α is also known, however, to upregulate exhaustion markers TIM-3 in CD8+ T cells induced by programmed cell death-1 (PD-1) antibody therapy [37]. TNF-α also plays an important role in metastasis by increasing the expression of angiogenic factors such as IL-8, vascular endothelial growth factor (VEGF) in endothelial cells of the TME, and basic fibroblast growth factor (bFGF).

TGF-β, a pleiotropic growth factor, under normal physiological conditions maintains homeostasis by inhibiting the growth of cells and stimulating apoptosis. However, the role of TGF-β in carcinogenesis is complex. TGF-β acts as a pro- or anti-tumorigenic factor depending on the stage of tumorigenesis. In the initial stages, TGF-β inhibits tumor growth due to cell-cycle blockade in cells undergoing transformation and during the later stages become pro-tumorigenic due to resistance developed to the anti-proliferative activity of TGF-β by tumor cells [38]. TGF-β is known to recruit Tregs and myeloid cells with a pro-tumorigenic polarization such as neutrophils, MDSCs, macrophages, and tolerogenic DCs and reduces NK cell and CD8+ T cell function [39].

### 4.2. T Cell Exhaustion in the TME

Naïve T cells transform to CD8+ effector T cells following antigen stimulation, which produces cytokines to kill tumor cells. Effector T cells either undergo apoptosis or differentiate over time into memory T cells. In the solid TME, T cells often express high levels of inhibitory receptors, lose their ability to produce IL-2, TNF-α, IFN-γ, and granzyme, and enter a state of exhaustion [40]. Constant exposure to a tumor antigen leads to the enhanced expression of inhibitory receptors such as PD-1, cytotoxic T lymphocyte antigen-4 (CTLA-4), T-cell immunoglobulin domain and mucin domain protein-3 (TIM-3), lymphocyte activation gene-3 (LAG-3), T-cell immunoglobulin and immunoreceptor tyrosine-based inhibitory motif domain (TIGIT), and band T lymphocyte attenuator (BTLA) [40,41]. T cell function can be restored by blocking these inhibitory receptors as validated by clinical successes of PD-1 and CTLA-4 antibodies. However, combination therapies targeting multiple inhibitory molecules might be required for the efficient revival of T cell function but needs to be optimized since excessive T cell function could lead to increased cytokine release and autoimmune reactions. Moreover, certain tumors are “cold” and lack a high degree of neo-antigen expression and T cell infiltration. In such situations, combinatorial approaches including agents such as toll-like receptor agonists may enable enhanced checkpoint responsiveness [42].

### 4.3. Novel Delivery Technologies

Off-target effects and high intra-tumoral pressure are significant issues associated with solid tumor immunotherapy treatment. The goal of novel delivery approaches in immunotherapy protocols is to enable the targeted and optimal delivery of therapies in tumors so that there are minimal off-target effects, optimizing the therapeutic index. Moreover, innovative delivery solutions may facilitate modulation of organ-specific immunosuppressive programs. Novel delivery strategies may involve device technologies or creative drug formulations. For example, liposomal nanoparticles complexed with a PD-L1 trap plasmid and cationic protamine to form lipid-protamine-DNA (LPD) nanoparticles have been tested as a method for targeting tumor tissue using aminoethyl anisamide ligands [43]. When mice bearing orthotopic colorectal tumors were injected intravenously with oxaliplatin and tumor targeted LPD, there was synergistic inhibition of tumor with reduced toxicity compared to mice treated with PD-L1 antibodies and oxaliplatin [43]. Also, nanomedicines can be designed to improve drug penetration at tumor sites [44]. In another study, 100 nm nanoparticles composed of gelatin were coated with 10 nm quantum dots that were released on exposure to matrix metalloproteinases, which are prevalent in TME [44]. In this study, nanoparticles were intra-tumorally injected into fibrosarcoma tumors in a dorsal skin-fold model, and the quantum dots that were delivered on nanoparticles had improved penetration into the tumor as compared to nonreactive quantum dots alone [44]. Local delivery using implantable scaffolds and intra-tumoral injections is desirable but is not always feasible for tumors that are not easily accessible. Regional delivery using specialized approaches designed to optimize therapeutic delivery through the modulation of pressure and flow have shown promise in pre-clinical models and clinical trials with a durable antitumor activity and low toxicity profile in solid tumors [45,46,47].

## 5. Immunotherapy Types for Solid Tumors

The fundamental goal of cancer immunotherapy is to stimulate the host immune system and re-engineer immune cells to target and eradicate tumor cells, ideally providing durable disease control. Unfortunately, the biologic challenges that limit patient endogenous anti-cancer immunity often suppress responsiveness to immunotherapy interventions as well. There is often an unfavorable imbalance in the TME between immune stimulatory and inhibitory pathways, whereby immunosuppression blocks effective immunotherapy responsiveness [48]. The TME chemokine and cytokine profile dictates the immune cell localization and can either promote or inhibit tumor development/progression, e.g., activating downstream transcription factors such as STAT, SMAD, AP-1, and NFκB and caspases, and cytokines control the pro- (TNFα, IL-6, IL-7, IL-23) or anti-tumorigenic (IL-12, IFNγ, TRAIL) activities [48]. At the immune cell level, lymphocytes such as NK, CD8+, and CD4+ helper T cells, and pro-inflammatory macrophage subtype M1 and DCs elicit an anti-tumor response while MDSCs and Tregs impede tumor immunity [48]. Favorable TME reprograming to support cellular immune function and limit immunosuppressive pathways may enable better outcomes in advanced solid tumor patients.

### 5.1. Checkpoint Inhibitors

Monoclonal antibodies (mAbs) are a type of cell therapy designed to target specific antigens present on tumor cells. Therapeutically attractive targets are the checkpoint inhibitor molecules, which include CTLA-4, PD-1, and its ligand, PD-L1 [49]. Immune checkpoints are a part of the immune system that prevents an excessive immune response that can destroy healthy cells in the body. Immune checkpoint signaling within T cells, for example, is initiated when the cognate ligand is engaged on tumor or suppressive immune cells. Checkpoint molecule activation results in downregulation of immune effector programs, which may culminate in reduced cytotoxic function and less favorable cytokine profiles. ICI block these checkpoint proteins from binding to their ligands, preventing the “off” signal. As previously stated, Ipilimumab was the first FDA-approved drug to block a checkpoint inhibitor, CTLA-4 [50,51]. Soon to follow were Nivolumab and Pembrolizumab, the first two anti–PD-1 mAbs that received FDA approval [52]. Checkpoints, like CTLA-4, PD-1, and PD-L1, are immune system regulators that are supposed to activate the immune response [53,54].

As of 2020, there are currently roughly 3000 ongoing clinical trials that are evaluating T cell modulators [52]. Many of these trials involve the PD-1/PD-L1 axis or CTLA-4. PD-1 is expressed on activated T cells, B cells, and myeloid cells, and its two ligands, PD-L1 and PD-L2, are expressed on tumor cells and MDSCs in liver metastasis murine tumor models [55,56]. The PD-1/PD-L1 axis causes immunosuppression by inducing apoptosis in activated T cells, facilitating T cell exhaustion and anergy, enhancing Treg immunosuppressive function, limiting T cell proliferation, and restraining T cell activation and IL-2 production [55]. Blocking PD-1 or PD-L1 inhibits the PD-1/PD-L1 interaction, which then signals T cells to kill cancer cells and create an immune response.

CTLA-4 is expressed on activated T cells and is known to regulate T cell proliferation as an early immune response as opposed to PD-1, which suppresses T cells as a late immune response—primarily in peripheral tissues [57]. Approved PD-1 inhibitors include Pembrolizumab (Keytruda), Nivolumab (Opdivo), Cemiplimab (Libtayo); PD-L1 inhibitors include Atezolizumab (Tecentriq), Avelumab (Bavencio), and Durvalumab (Imfinzi); and a CTLA-4 inhibitor includes Ipilimumab (Yervoy). An anti-CTLA-4 (ipilimumab) and anti-PD-1 (nivolumab and pembrolizumab) combination has been studied extensively in metastatic melanoma patients and the efficacy of the combination was demonstrated in multiple clinical trials [58]. In a phase 1 study, an ipilimumab and nivolumab combination was reported to increase the objective response rate to 61% (*n* = 44/72), with complete responses seen in 22% (*n* = 16/72) of patients [59]. Patients in this study reportedly had significantly lower incidence of disease progression or death. In another phase 2 study, patients with this combination therapy had an increase in the 2-year overall survival to 63.8% [60]. In the phase 3 study, patients treated with nivolumab plus ipilimumab, compared to ipilimumab or nivolumab monotherapy, had a higher objective response rate (57%, 19%, and 44%, respectively), longer median progression free survival (PFS, 11.5, 2.9, and 6.9 months, respectively), and lower incidence of disease progression or death [61]. Results of the outcomes after 3-year and 4-year follow-up of patients pointed towards the superior clinical benefits of combination therapy over monotherapy [62,63]. They have helped improve clinical patients with different types of cancers, including breast, bladder, cervical, colon, liver, and lung [64]. Having the ability to impact such a wide range of cancers is a considerable leap for immunotherapy. Although there are currently no precise biomarkers to predict the response of ICI, its efficacy, in general, is associated with the expression of (PD-1, PD-L1, and CTLA-4) and tumor mutation burden [55,65]. Detecting therapeutic response, prognostic biomarkers, and utilizing a combination of two or more ICIs can significantly improve their efficacy.

A mechanism to overcome the limited efficacy of PD-1/PDL-1 ICIs is to target other TME-associated immune checkpoint molecules, such as TIM-3 and LAG-3. A phase 2 study conducted on 72 patients treated with LAG-3 IgG4 mAb (LAG-525) and an anti-PD-1 (spartalizumab) antibody for advanced solid tumors and hematologic malignancies showed promising results, especially in neuroendocrine tumors, small cell lung cancer, and diffuse large B-cell lymphoma. The anti-LAG-3/PD-1 combination demonstrated a clinical benefit rate at 24 weeks of 86%, 27%, and 81%, respectively, in the indications mentioned above (NCT03365791) [66]. There are seven anti-TIM-3 monoclonal antibodies and one anti-PD-1 and TIM-3 bispecific Ab (RO7121661) undergoing clinical development. Sym021 (anti-PD-1), sym022 (anti-LAG-3), and sym023 (anti-TIM-3) were evaluated as single or combination treatments in phase 1 trials for solid tumors or lymphomas (NCT03311412, NCT03489369, and NCT03489343) [67]. The monotherapy and combination therapy were well tolerated with two partial responses observed in the combination group. Overall, a multi-checkpoint inhibition strategy appears rational, given the presence of redundancy and biologic complexity in most solid organ TMEs.

### 5.2. Bi-Specific Antibodies

Bispecific monoclonal antibodies (BsAb) are genetically engineered recombinant antibodies that can simultaneously target two antigens. To date, two BsAbs, blinatumomab and emicizumab, have been approved in the US and the EU. Currently, more than 60 BsAb drugs are in pre-clinical trials and 30 are in clinical trials. Two-thirds of the BsAbs focus on the treatment of cancer by bringing effector T cells closer to cancer cells that express specific surface antigens (BiTE) [68]. BiTEs only trigger T cell cytotoxicity and cytokine production when both antigen binding sites are occupied [69] and is known to preferentially activate memory T cells [70,71,72]. BiTEs are small in size and rapidly penetrate tumors and tissues, however they also get quickly cleared by kidneys and continuous dosing is required [73,74].

The BiTE blinatumomab has demonstrated clinical response at very low doses in patients with non-Hodgkin lymphoma as compared to intact antibodies such as rituximab (anti-CD20) [74]. The side effects specific to BiTEs are neurotoxicity and CRS, which was observed in the first FDA-approved mAb, blinatumomab [74]. Emicizumab, a humanized bispecific antibody that binds to both activated coagulation factors IX and X, was approved for treatment of acquired hemophilia A, a severe bleeding disorder caused by inhibitory auto-antibodies against coagulation factor VIII [68]. BsAbs are in development and a proper understanding of protein engineering and design to create these molecules with proper delivery strategy is crucial to overcome treatment related adverse events. BiTEs likely will require the presence of T cell infiltrates within tumors to mediate their mechanism of action. As such, immunologically “cold” tumors with a negligible lymphocyte presence may pose significant challenges for this class of drug.

### 5.3. Oncolytic Viruses (OV)

Cancer cells have impaired antiviral defenses, which makes them vulnerable to OV. After infection, OVs cause lysis of cancer cells, thereby releasing the antigens and stimulating the immune response towards the remaining tumor cells. It was observed in the 19th century that some cancer patients would go into a small state of remission, most notably leukemia patients who contracted influenza [75,76]. OVs work in two different ways: they can be selected, to target, replicate in, and lyse tumor cells while avoiding healthy tissue and/or induce an immune response to have the body’s innate immunity do the killing [77]. Although there is a push to use genetically engineered OVs, some naturally occurring viruses, such as Reolysin, a proprietary variant of the non-pathogenic reovirus that naturally resides in the digestive or respiratory tract, could be used to combat cancer as well [78]. T-Vec (Imlygic), an attenuated herpesvirus encoding granulocyte-macrophage colony stimulating factor (GM-CSF), is the only FDA-approved OV for treating melanoma patients [79]. GM-CSF can enhance the inflammatory response by activating immune cells and is used as an immunostimulant in cancer therapies, as mentioned above. However, tumor-derived GM-CSF, such as in pancreatic cancer and in liver metastases, cause the activation and expansion of immunosuppressive MDSC, which can suppress effector T cell functions [26,56,80]. Hence, organ and disease-specific biology should be taken into account to better identify patients who will be likely to respond to treatment and to decipher potential mechanisms of treatment resistance.

### 5.4. Cancer Vaccines

Dr. William B. Coley’s “toxins” paved the way for the modern study of cancer immunology. Observing the effects of fever on sarcoma patients, Dr. Coley started inoculating his patients with Streptococcus pyogenes and Serratia marcescens. Johnston et al. would later validate Coley’s work and bring cancer vaccines into the mainstream. Cancer vaccines are categorized as cellular, viral vector, or molecular (DNA, peptide, or RNA) vaccines. Cellular vaccines are developed from an autologous or allogeneic tumor cell line. Dendritic cells are used to develop cellular cancer vaccines due to their roles as antigen-producing cells. Viral vector vaccines promote tumor-directed immune responses by delivering antigens via T-cell priming [81]. Also, mRNA vaccines encode antigens that express proteins following internalization, which causes an immune response. These vaccines can deliver high numbers of antigens with a low risk of infection or insertional mutagenesis. In general, vaccines for solid tumors may often face the challenge of TMEs already programmed in a highly immunosuppressive manner, which limits the ability of patients to develop meaningful anti-tumor responses to tumor antigens.

#### 5.4.1. Preventative vs. Therapeutic Vaccines

Cancer vaccines that aim to reduce cancer incidence, morbidity, and mortality are termed preventative or prophylactic. These vaccines have found success in the primary prevention of cancers, secondary to both HBV (Hepatitis B virus) and HPV (Human Papillomavirus). In contrast, therapeutic cancer vaccines are utilized to treat an existing disease or prevent relapse or metastases [82]. Therapeutic cancer vaccines accomplish this by producing responses directed against antigens specific to tumors with the goal to activate the immune system through antigen presentation [83]. A challenge in developing cancer vaccines is the possibility that the host has already become tolerant to the targeted tumor-associated antigens based on suppressive TME programming.

#### 5.4.2. Clinical Trials

Therapeutic cancer vaccine clinical trials have seen few successes. Only a handful of vaccines have been approved in the US or EU, while several phase 3 clinical trials have failed to deliver results leading to discontinuation [83]. The successful products are BCG (TheraCys & TICE), Sipuleucel-T (PROVENGE), and T-Vec (IMLYGIC) [84]. BCG, a vaccine to prevent and treat urothelial carcinoma, became FDA approved in 1990 in the form of TheraCys. A phase 3 trial treating patients with intravesical BCG vaccine demonstrated a 5-year disease-free survival of 45% [85]. Sipuleucel-T, an autologous cellular immunotherapy for metastatic castrate-resistant prostate cancer, was approved in 2010 [84]. GVAX vaccines are genetically modified tumor cells that can secrete immune stimulatory GM-CSF. Several clinical trials testing GVAX vaccines in melanoma, pancreatic cancer, lung cancer, and prostate cancer have shown limited efficacy [86,87,88,89]. Combination therapies, including ICI that reverse immunosuppression with cancer, may improve the likelihood of success. Cancer vaccine progress lies in the identification of multiple immunogenic antigens, generating potent vaccine vectors, and overcoming solid tumor-mediated immunosuppression.

### 5.5. Adoptive Cell Therapy (ACT)

**Figure 1:**
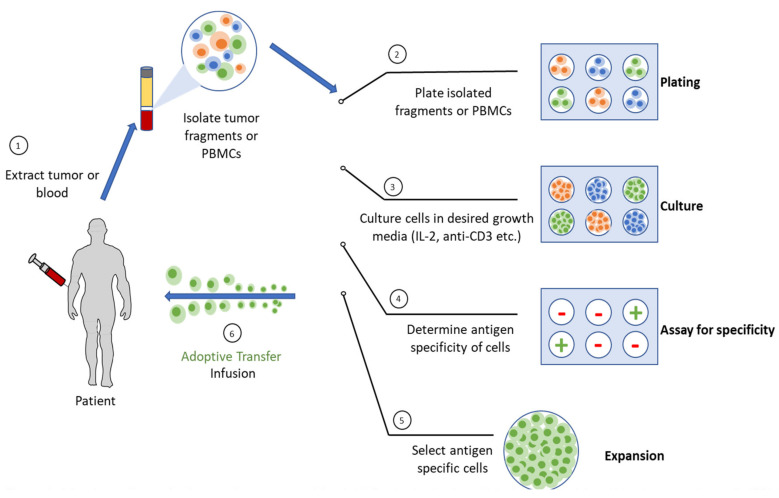
Adoptive cell transfer from patient tumor or blood. (1) Production begins with isolation of peripheral blood mononuclear cells (PBMC) from leukapheresis or tumor is excised and multiple individual cultures are isolated and (2) plated separately followed by (3) selection and activation of T cells. (4) T cells then undergo genetic modification for generating CAR-T cells or tumor cultures are assayed for specific tumor recognition. (5) Cells are expanded in presence of interleukins and when desired dose cell numbers are achieved, expanded cells are harvested and dose is formulated. (6) QC tests are performed to ensure that drug meets release criteria and is then fused into patients with or without conditioning lymphodepleting chemotherapy (6).

ACT is another form of cell therapy that uses autologous or allogeneic immune cells to eliminate cancer. The patient’s own (autologous) immune cells can be isolated, bioengineered for tumor antigen specificity, expanded, and reinfused into the patient, as shown in Figure 1. The ability to isolate and expand immune cells ex vivo offers the advantages of being able to select immune cells with high-avidity recognition and effector function, expand them to large numbers in the absence of inhibitory factors that exist in vivo, and the ability to manipulate the host’s TME before the infusion to better support immune cell function and persistence [90].

#### 5.5.1. Autologous Cell Therapy

The driving principle of ACT—that certain immune cells have enhanced antitumor function—is predominantly provided by Cytotoxic T Lymphocyte (CTL) properties and its T-cell receptor (TCR). The CTL is the immune effector cell primarily responsible for combating solid tumors, and its TCR provides the specificity for certain target antigens. When tumor antigens are presented to T cells, their TCRs become primed to target these tumors specifically, allowing for enhanced immune responses [91]. TILs are examples of these enhanced T cells that can recognize tumor-associated antigens to target and eliminate tumors specifically. These TILs can be isolated from the excised tumor, then be activated ex vivo and expanded up to 100 billion cells [92]. Prior to infusion, patients may be lymphodepleted, which has demonstrated an improvement of ACT treatment efficacy [93]. In addition to impressive response rates, longer response durability has also been reported in clinical trials [94,95]. Using a patient’s own T cells greatly limits the risk of developing an immunological reaction against donor cells and of graft-versus-host disease (GvHD). 

CAR-T are T cells that have been genetically engineered to express specialized receptors that offer the immune cell enhanced performance [96]. These receptors have several components, such as an extracellular binding domain, a hinge region, a transmembrane domain, and intracellular signaling domains [97]. In general, a monoclonal antibody, the single chain variable fragment (scFv) is utilized as the extracellular binding domain, providing CAR-T with tumor antigen specificity. This selectivity, coupled with T cells’ immunological properties, makes CAR-T therapy a potent antitumor treatment [96]. 

**Figure 2:**
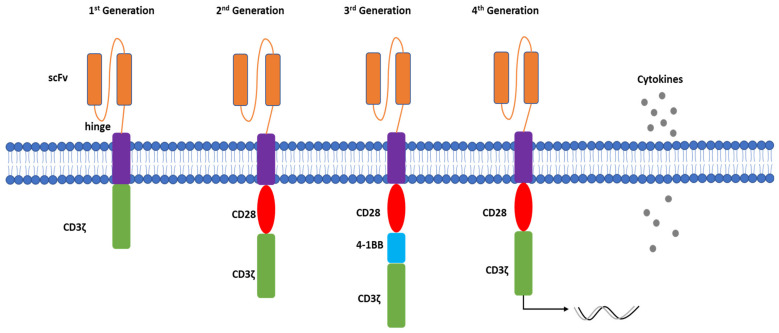
Four generations of CAR-T cells. First generation CARs comprise of single chain variable fragment (scFv) antibody (orange) fused to transmembrane domain (purple) to TCR signaling component of CD3ζ (green) at the cytoplasmic tail. Second generation CARs have a CD28 co-stimulatory signaling domain (red) which enhances proliferation and cytotoxicity. Third generation CARs contain an additional co-stimulatory domain, 4-1BB (blue) to the second generation CARs which enhances proliferation, minimizes T cell exhaustion and improves CAR-T cells persistence. Fourth generation CARs also called “T cell directed for universal cytokine-mediated killing” (TRUCKs) are engineered to secrete cytokines (gray) to attract immune cells (NK and macrophages).

CAR-T therapy is continually evolving, improving its function with each successive generation, as shown in Figure 2. The first generation of CAR-T comprises of a single structure of the CD3ζ chain, which is known to have limited CAR proliferation and tumor-killing efficacy. The second and third generation significantly improved CAR-T function with one or two co-stimulatory domains, such as CD28 or 4-1BB. The second signal from the co-stimulatory domain promotes IL-2 synthesis, which activates T cells and enhances their proliferation and in vivo persistence [97]. Fourth-generation CAR-T, also referred to as armored CARs or T cells redirected for universal cytokine-mediated killing (TRUCKs), are enhanced with factors that improve the antitumor function, such as cytokines or enzymes [98]. To enhance the safety of CAR-T therapy, CAR-T with a “safety switch” of inducible Caspase 9 (iCasp9) has been designed [99]. This allows for the termination of inappropriately activated CAR-T cells, preventing potential immunopathology [98]. Table 1 lists the clinical trials in the USA of CAR-T cells against most commonly targeted tumor antigens.

#### 5.5.2. Allogeneic Cell Therapy

Allogeneic cell therapy offers several advantages over autologous cell therapy but also presents different challenges. The “off-the-shelf” aspect of allogeneic cell therapy saves patients valuable time by eliminating the long “vein-to-vein” lag encountered with autologous products. Since cells originate from healthy donors who are not exposed to chemotherapy, the risk of production failure may be lower. Also, there is consistency between the doses as they are generated from the same donor and hence are considered the same lot. The complex production of doses can be performed in a more controlled way using standard quality assurance with the option of scaling up the manufacturing process, thereby contributing to lower production costs. Unfortunately, rejection, GvHD, and poor persistence are challenges in the allogeneic ACT space. There are several variants of allogeneic ACT products, which we discuss below.

Building on the success of CAR-T cells, CARs have also been applied to NK cells. While CAR-T therapy has proven effective in treating hematological and solid tumors, CAR-NK cells have certain advantages in a few areas. Unlike T cells, NK cells are not bound by HLA restrictions [100]. This property allows CAR-NK to be used in an allogeneic setting, with the potential of becoming a universal “off-the-shelf” cellular therapy product [101]. It has been demonstrated clinically that CAR-NK can survive in vivo for several weeks to months [102]. Also, CAR expression on NK cells allows for more effective cytotoxicity in solid tumors compared to normal NK-mediated cytotoxicity, as they inherently possess tumor cytotoxicity and can be activated independent of CAR mechanisms via natural cytotoxicity receptors (NCRs)—such as NKp30, NKp44, and NKp46, and DNAM-1 co-stimulatory receptor and NKG2D [103].

NK cells can also be derived from several sources, such as cord blood, induced pluripotent stem cells (iPSCs), and PBMCs [104]. In an early clinical trial (NCT03383978) NK cell line, NK-92 is currently being tested. NK cells generated from PBMCs or cord blood relies on the use of irradiated feeder cells, such as K562. iPSCs present an alternative source for generating NK cells without the feeder cells [105]. Also, iPSCs can be genetically modified prior to NK cell differentiation, thereby enabling the provision of an unlimited supply of NK cells [106].

Allogeneic γδ T cells play an important role in tissue homeostasis and cancer immunosurveillance. These cells have been infused into patients after lymphodepleting chemotherapy and were shown to expand in vivo without causing GvHD [107]. γδ T cells are abundant in tissues and may have an edge over αβ T cells for developing therapeutic strategies for treating solid tumors [108]. Polyclonal γδ T cells transduced with CD19 CAR and GD2 CAR have demonstrated anti-tumor effects in vitro and in in vivo murine models [109,110].

Despite the potential benefits of allogeneic cell therapy, there are significant challenges that need to be overcome for their successful implementation. Some of the challenges include an immunologic mismatch between donor and recipient that may cause undesirable GvHD. Conversely, if a recipient’s immune system recognizes and reacts to allogeneic therapy, the dose may be rejected, thereby limiting its therapeutic activity. It is likely that autologous and allogeneic ACT products will both find a place within the multi-disciplinary management of solid tumor patients. The relative value of autologous and allogeneic therapy in specific clinical settings will be determined by disease biology, typical patient physiology, and the existing therapeutic landscape.

#### 5.5.3. Combination Therapy or Other Strategies of Regional Delivery

To increase the likelihood of success with immunotherapy in challenging solid tumor indications, a variety of combinatorial approaches are being explored. Multiple clinical studies have shown that CAR-T therapy or chemotherapy alone achieved limited efficacy to treat solid tumors [111,112]. However, studies have found that many chemotherapeutic agents, such as cyclophosphamide, doxorubicin, oxaliplatin, fluorouracil, and gemcitabine, reduce tumor burden and have immunomodulatory effects that enhance immunotherapy [113]. Radiotherapy has been known to react synergistically with CAR-T therapy. Not only does radiotherapy eradicate the tumor, but it can also sensitize tumor cells to cytotoxic lymphocytes in the murine colon adenocarcinoma model [114], modulate the TME to make it conducive to CAR-T infiltration, traffic in the highly angiogenic insulinoma mouse models [115], and improve tumor antigen presentation as observed in tumors implanted in flanks of mice [116].

CAR-T treatment can be combined with other immunotherapies as well. Tumors often employ their immunosuppressive microenvironments to escape the therapeutic effects of CAR-T therapy [117]. Antibodies that block CTLA-4, PD-1, and PD-L1 can be used to overcome the immunosuppressive nature of the TME and allow the CAR-T to maintain efficacy [118]. Combinations of CAR-T with chemokines such as CXCR2 or CCR4 have been found to help CAR-T trafficking and persistence in solid tumors [118]. It has also been found that combining CAR-T therapy with OVs produces synergistic effects. OVs can assist in the entry of CAR-T into tumors [119]. CAR-T has also been shown to better survive in solid tumors in the presence of OVs [120]. 

#### 5.5.4. Side Effects

Although CAR cellular therapy has proven highly beneficial in treating tumors, these results are often accompanied by potential toxic side effects. Overcoming these side effects is paramount to the successful application of these cell therapies. The most prevalent of these side effects is CRS, or elevated inflammatory cytokines resulting from therapy-induced immune activation. CRS can range from mild to severe, with the latter being potentially life-threatening. Symptoms can include high fever, malaise, fatigue, myalgia, nausea, anorexia, tachycardia/hypotension, capillary leak, cardiac dysfunction, renal impairment, and hepatic failure disseminated intravascular coagulation [121].

Another potential side effect is OTOTT in CAR cellular therapy, which can be avoided only in cases where the target antigen is exclusively restricted to the tumor. Unfortunately, many CAR cells target antigens are shared on normal tissue, resulting in OTOTT [122]. On-target/off-tumor recognition is often observed in the gastrointestinal, hematologic, and pulmonary organ systems [123]. Targeting colon cancer with CAR therapy using the carcinoembryonic antigen (CEA) has caused severe colitis due to recognition of the normal colon tissue [124]. Anaphylaxis is another concern when treating patients with CAR cells due to the murine mAb origin of the antigen-recognition domains [122]. These domains can be recognized as a foreign protein and elicit an immune response [125].

## 6. Summary

The major roadblock for ACT is to translate this treatment modality to solid malignancies to treat solid tumors effectively and to improve its survival, persistence, and efficacy. While autologous treatments such as CAR-T cell therapy have proven effective, HLA restrictions prevent the drug’s universal application. This also makes the manufacturing of cellular therapy incredibly expensive and time-consuming, which is problematic for some patients with highly proliferative diseases, as it can result in disease progression. While CAR-NK cells do not face these same HLA restrictions, this therapy is more novel and requires further exploration. The development of a universal “off-the-shelf” cellular therapy would revolutionize immunotherapy. 

The immunosuppressive TME of the solid tumor remains a major obstacle to overcome, with the potential for variation among anatomic sites. Combination therapies with checkpoint blockade, vaccines, OVs, cell therapies, etc., have shown that these different methods have the potential to counter immunosuppression more effectively while also providing direct tumor killing activity in some cases. Further combinations are required to address the difficulties presented by solid tumors. Novel delivery methods are also worthy of consideration to optimize the therapeutic concentration in solid tumors and to overcome delivery barriers imposed by physical forces within the TME. High-pressure delivery of CAR-T cellular therapy has shown increased penetration and persistence of cells in tumors [45,46]. Regional delivery of cell therapy may also enhance the therapeutic index by limiting systemic toxicity, including CRS and neurologic side effects.

## 7. Conclusions and Perspectives

Although immunotherapy has shown great promise in certain indications, ICIs and cell therapy have thus far failed to make a major impact on certain solid tumor indications. CAR-T cell therapy has completely revolutionized patient care for some hematologic malignancies, but has yet to achieve similar levels of success in the realms of solid organ primary or metastatic malignancies. An unprecedented number of clinical trials of CAR-T cells in solid tumors are ongoing with novel strategies creating increased levels of optimism. Barriers to immunotherapy success in solid tumors such as CAR-T cell trafficking, persistence, immunosuppressive TME, and antigen heterogeneity have been identified. Hence, increased understanding of the critical interactions between tumors and immune responses is imperative. Combinatorial approaches specifically tailored to both the disease and organ biology hold the greatest promise in extending the positive impact of immunotherapy to a greater number of patients in need. Also, the development of specific biomarkers to delineate the use of the optimal immunotherapy is critical to ensure the best treatment option for patients. Overall, novel cancer immunotherapies have revolutionized cancer treatment for patients by enhancing clinical outcomes for patients and results from ongoing research should also assist in its continued progress in the field.

## Figures and Tables

**Table 1:** Commonly targeted solid tumor antigens in clinical trials in USA.

Antigen	Cancer	Phase	Trial ID #
CD20	Melanoma, Non Hodgkin Lymphoma, Mantle cell Lymphoma	Phase 1/2	NCT04160195, NCT04186520
CD171	Neuroblastoma	Phase 1	NCT02311621
CEA	Lung, colorectal, gastric, breast, pancreatic, peritoneal, liver	Phase 1	NCT03682744, NCT01373047,
			NCT03818165, NCT02416466,
			NCT02850536
Claudin 18.2	Gastric, pancreatic	Phase 1	NCT04404595
EGFRIII	Glioblastoma, gliosarcoma and brain tumor	Phase 1/2	NCT01454596, NCT03283631
EGFR806	Central nervous system tumor, pediatric glioma	Phase 1	NCT03638167, NCT03618381
GD2	Glioma	Phase 1	NCT04099797
Glypican-3	Liver	Phase 1	NCT02932956, NCT02905188
			NCT04377932
HER2	Central nervous system tumor, pediatric glioma, breast	Phase 1/2	NCT03500991, NCT03696030
			NCT02442297, NCT03740256
			NCT04483778, NCT03618381
			NCT00924287, NCT04650451
			NCT01109095
HLA-A2	Ependymoma	Phase 1	NCT01795313
IL-13Rα2	Glioblastoma, cutaneous melanoma	Phase 1	NCT02208362, NCT04119024
			NCT04003649
KK-LC-1	Epithelial	Phase 1	NCT05035407
Mesothelin	Ovarian, cervical, pancreatic, lung, breast, mesothelioma	Phase 1/2	NCT01583686, NCT04577326
			NCT03054298, NCT02159716
			NCT02414269
PSCA	Prostate cancer, metastatic pancreatic	Phase 1/2	NCT03873805, NCT02744287
TAA-T	Hematopoietic malignancies, acute myeloid leukemia, MDS, Hodgkin lymphoma, B cell lymphoma	Phase 1	NCT02203903, NCT03843294
VEGFR2	Metastatic melanoma, renal	Phase 1/2	NCT01218867

## Data Availability

Not applicable.

## Abbreviations

ACT	adoptive cell therapy
ADCs	antibody-drug conjugated
AP-1	activator protein 1
bFGF	basic fibroblast growth factor
BsAb	bispecific monoclonal antibodies
BCMA	B-cell maturation antigen
BsAb	bispecific antibody
BiTEs	bispecific T cell engagers
BTLA	band T lymphocyte attenuator
CAR-NK	chimeric antigen receptor NK cells
CAR-T	chimeric antigen receptor T cells
CRS	cytokine release syndrome
CTLA-4	cytotoxic T lymphocyte antigen 4
FDA	food and drug administration
GvHD	graft-versus-host disease
HBV	hepatitis B virus
HPV	human papillomavirus
HLA	human leukocyte antigen
iCasp9	inducible Caspase 9
ICI	immune checkpoint inhibitors
IFN	interferon
IL	interleukin
iPSC	induced pluripotent stem cells
LAG-3	lymphocyte activation gene 3 protein
LPD	lipid-protamine-DNA
mAb	monoclonal antibody
MDSCs	myeloid derived suppressor cells
NF-κB	nuclear factor kappa B
mRNA	messenger RNA
OTOTT	on-target-off-tumor-toxicity
OV	oncolytic virus
PD-1	programmed cell death protein
PD-L1	programmed cell death protein and its ligand
scFv	single chain variable fragment
STAT3	signal transducer and activator of transcription 3
TAM	tumor associated macrophages
TGF-β	transforming growth factor beta
TIL’s	tumor-infiltrating lymphocytes
TIM-3	T-cell immunoglobulin domain and mucin domain protein 3
TME	tumor microenvironment
Tregs	regulatory T cells
TIGIT	T-cell immunoglobulin and immunoreceptor tyrosine-based inhibitory motif domain
TNF	tumor necrosis factor
TRAIL	TNF related apoptosis inducing ligand
VEGF	vascular endothelial growth factor

## References

[1] Kucerova P., Cervinkova M. (2016). Spontaneous regression of tumour and the role of microbial infection—Possibilities for cancer treatment. Anti-Cancer Drugs.

[2] Dobosz P., Dzieciątkowski T. (2019). The Intriguing History of Cancer Immunotherapy. Front. Immunol..

[3] Mellman I., Coukos G., Dranoff G. (2011). Cancer immunotherapy comes of age. Nature.

[4] Burga R.A., Thorn M., Point G.R., Guha P., Nguyen C.T., Licata L.A., DeMatteo R.P., Ayala A., Espat N.J., Junghans R.P. (2015). Liver myeloid-derived suppressor cells expand in response to liver metastases in mice and inhibit the anti-tumor efficacy of anti-CEA CAR-T. Cancer Immunol. Immunother..

[5] Swanson G.P., Rynearson K., Symmonds R. (2002). Significance of margins of excision on breast cancer recurrence. Am. J. Clin. Oncol..

[6] DeVita V.T., Chu E. (2008). A History of Cancer Chemotherapy. Cancer Res..

[7] De Angelis C. (2008). Side Effects Related to Systemic Cancer Treatment: Are We Changing the Promethean Experience with Molecularly Targeted Therapies?. Curr. Oncol..

[8] Baskar R., Lee K.A., Yeo R., Yeoh K.-W. (2012). Cancer and Radiation Therapy: Current Advances and Future Directions. Int. J. Med. Sci..

[9] Cheng H., Wang Z., Fu L., Xu T. (2019). Macrophage Polarization in the Development and Progression of Ovarian Cancers: An Overview. Front. Oncol..

[10] Jiang T., Zhou C. (2015). The past, present and future of immunotherapy against tumor. Transl. Lung Cancer Res..

[11] Ventola C.L. (2017). Cancer Immunotherapy, Part 1: Current Strategies and Agents. Pharm. Ther..

[12] Filin I.Y., Solovyeva V.V., Kitaeva K.V., Rutland C.S., Rizvanov A.A. (2020). Current Trends in Cancer Immunotherapy. Biomedicines.

[13] Maeda H., Khatami M. (2018). Analyses of repeated failures in cancer therapy for solid tumors: Poor tumor-selective drug delivery, low therapeutic efficacy and unsustainable costs. Clin. Transl. Med..

[14] Siegel R.L., Miller K.D., Goding Sauer A., Fedewa S.A., Butterly L.F., Anderson J.C., Cercek A., Smith R.A., Jemal A. (2020). Colorectal cancer statistics, 2020. CA Cancer J. Clin..

[15] Hou B., Tang Y., Li W., Zeng Q., Chang D. (2019). Efficiency of CAR-T Therapy for Treatment of Solid Tumor in Clinical Trials: A Meta-Analysis. Dis. Markers.

[16] Duffy A.G., Greten T.F. (2013). Immunological off-target effects of standard treatments in gastrointestinal cancers. Ann. Oncol..

[17] Giampieri R., Del Prete M., Prochilo T., Puzzoni M., Pusceddu V., Pani F., Maccaroni E., Mascia R., Baleani M.G., Meletani T. (2017). Off-target effects and clinical outcome in metastatic colorectal cancer patients receiving regorafenib: The TRIBUTE analysis. Sci. Rep..

[18] Brown J.M., Giaccia A.J. (1998). The unique physiology of solid tumors: Opportunities (and problems) for cancer therapy. Cancer Res..

[19] Khawar I.A., Kim J.H., Kuh H.-J. (2015). Improving drug delivery to solid tumors: Priming the tumor microenvironment. J. Control Release.

[20] Vaupel P., Thews O., Hoeckel M. (2001). Treatment resistance of solid tumors: Role of hypoxia and anemia. Med. Oncol..

[21] Trédan O., Galmarini C.M., Patel K., Tannock I.F. (2007). Drug Resistance and the Solid Tumor Microenvironment. J. Natl. Cancer Inst..

[22] Chai L.F., Prince E., Pillarisetty V.G., Katz S.C. (2019). Challenges in assessing solid tumor responses to immunotherapy. Cancer Gene Ther..

[23] Whiteside T.L. (2008). The tumor microenvironment and its role in promoting tumor growth. Oncogene.

[24] Poggi A., Musso A., Dapino I., Zocchi M.R. (2014). Mechanisms of tumor escape from immune system: Role of mesenchymal stromal cells. Immunol. Lett..

[25] Lindau D., Gielen P., Kroesen M., Wesseling P., Adema G.J. (2012). The immunosuppressive tumour network: Myeloid-derived suppressor cells, regulatory T cells and natural killer T cells. Immunology.

[26] Guha P., Gardell J., Rabinowitz B., Lopes M., DaSilva N.A., Rowley D., Katz S.C. (2020). Monocytic and granulocytic myeloid-derived suppressor cell plasticity and differentiation are organ-specific. Oncogene.

[27] Grivennikov S.I., Greten F.R., Karin M. (2010). Immunity, inflammation, and cancer. Cell.

[28] Yu H., Pardoll D., Jove R. (2009). STATs in cancer inflammation and immunity: A leading role for STAT3. Nat. Cancer.

[29] Grivennikov S., Karin E., Terzic J., Mucida D., Yu G.Y., Vallabhapurapu S., Scheller J., Rose-John S., Cheroutre H., Eckmann L. (2009). IL-6 and Stat3 are required for survival of intestinal epithelial cells and development of colitis-associated cancer. Cancer Cell.

[30] Becker C., Fantini M.C., Schramm C., Lehr H.A., Wirtz S., Becker A., Burg J., Strand S., Kiesslich R., Huber S. (2004). TGF-beta suppresses tumor progression in colon cancer by inhibition of IL–6 trans-signaling. Immunity.

[31] Bollrath J., Phesse T., von Burstin V.A., Putoczki T., Bennecke M., Bateman T., Nebelsiek T., Lundgren-May T., Canli Ö., Schwitalla S. (2009). gp130-Mediated Stat3 Activation in Enterocytes Regulates Cell Survival and Cell-Cycle Progression during Colitis-Associated Tumorigenesis. Cancer Cell.

[32] Popivanova B.K., Kitamura K., Wu Y., Kondo T., Kagaya T., Kaneko S., Oshima M., Fujii C., Mukaida N. (2008). Blocking TNF-α in mice reduces colorectal carcinogenesis associated with chronic colitis. J. Clin. Investig..

[33] Wang D.J., Ratnam N.M., Byrd J.C., Guttridge D.C. (2014). NF-kappaB functions in tumor initiation by suppressing the surveillance of both innate and adaptive immune cells. Cell Rep..

[34] Wang Y., Shen Y., Wang S., Shen Q., Zhou X. (2018). The role of STAT3 in leading the crosstalk between human cancers and the immune system. Cancer Lett..

[35] Van Horssen R., Hagen T.L.M.T., Eggermont A.M.M. (2006). TNF-α in Cancer Treatment: Molecular Insights, Antitumor Effects, and Clinical Utility. Oncologist.

[36] Zhao L., Ching L.-M., Kestell P., Baguley B.C. (2002). The antitumour activity of 5,6-dimethylxanthenone-4-acetic acid (DMXAA) in TNF receptor-1 knockout mice. Br. J. Cancer.

[37] Bertrand F., Montfort A., Marcheteau E., Imbert C., Gilhodes J., Filleron T., Rochaix P., Andrieu-Abadie N., Levade T., Meyer N. (2017). TNFalpha blockade overcomes resistance to anti-PD-1 in experimental melanoma. Nat. Commun..

[38] Massagué J. (2008). TGFβ in cancer. Cell.

[39] Berraondo P., Sanmamed M.F., Ochoa M.C., Etxeberria I., Aznar M.A., Pérez-Gracia J.L., Rodriguez-Ruiz M.E., Ponz-Sarvise M., Castañón E., Melero I. (2019). Cytokines in clinical cancer immunotherapy. Br. J. Cancer.

[40] Jiang Y., Li Y., Zhu B. (2015). T-cell exhaustion in the tumor microenvironment. Cell Death Dis..

[41] Zarour H.M. (2016). Reversing T-cell Dysfunction and Exhaustion in Cancer. Clin. Cancer Res..

[42] Ribas A., Medina T., Kummar S., Amin A., Kalbasi A., Drabick J.J., Barve M., Daniels G.A., Wong D.J., Schmidt E.V. (2018). SD-101 in Combination with Pembrolizumab in Advanced Melanoma: Results of a Phase Ib, Multicenter Study. Cancer Discov..

[43] Song W., Shen L., Wang Y., Liu Q., Goodwin T.J., Li J., Dorosheva O., Liu T., Liu R., Huang L. (2018). Synergistic and low adverse effect cancer immunotherapy by immunogenic chemotherapy and locally expressed PD-L1 trap. Nat. Commun..

[44] Wong C., Stylianopoulos T., Cui J., Martin J., Chauhan V.P., Jiang W., Popović Z., Jain R.K., Bawendi M.G., Fukumura D. (2011). Multistage nanoparticle delivery system for deep penetration into tumor tissue. Proc. Natl. Acad. Sci. USA.

[45] Katz S.C., Hardaway J., Prince E., Guha P., Cunetta M., Moody A., Wang L.J., Armenio V., Espat N.J., Junghans R.P. (2019). HITM-SIR: Phase Ib trial of intraarterial chimeric antigen receptor T-cell therapy and selective internal radiation therapy for CEA+ liver metastases. Cancer Gene Ther..

[46] Katz S.C., Moody A.E., Guha P., Hardaway J.C., Prince E., Laporte J., Stancu M., Slansky J.E., Jordan K.R., Schulick R.D. (2020). HITM-SURE: Hepatic immunotherapy for metastases phase Ib anti-CEA CAR-T study utilizing pressure enabled drug delivery. J. Immunother. Cancer.

[47] Narayanan J.S.S., Vicente D.A., Ray P., Chai L.F., Erdem S., Carr M.J., Capacio B.A., Cox B.F., Jaroch D.B., Katz S.C. (2020). Pressure-enabled delivery of gemcitabine in an orthotopic pancreatic cancer mouse model. Surgery.

[48] Labani-Motlagh A., Ashja-Mahdavi M., Loskog A. (2020). The Tumor Microenvironment: A Milieu Hindering and Obstructing Antitumor Immune Responses. Front. Immunol..

[49] Pento J.T. (2017). Monoclonal Antibodies for the Treatment of Cancer. Anticancer Res..

[50] Graziani G., Tentori L., Navarra P. (2012). Ipilimumab: A novel immunostimulatory monoclonal antibody for the treatment of cancer. Pharmacol. Res..

[51] Lipson E.J., Drake C.G. (2011). Ipilimumab: An Anti-CTLA-4 Antibody for Metastatic Melanoma. Clin. Cancer Res..

[52] Robert C. (2020). A decade of immune-checkpoint inhibitors in cancer therapy. Nat. Commun..

[53] Andrews L.P., Yano H., Vignali D.A.A. (2019). Inhibitory receptors and ligands beyond PD-1, PD-L1 and CTLA-4: Breakthroughs or backups. Nat. Immunol..

[54] Quezada S., Peggs K.S. (2013). Exploiting CTLA-4, PD-1 and PD-L1 to reactivate the host immune response against cancer. Br. J. Cancer.

[55] Meyers D.E., Bryan P.M., Banerji S., Morris D.G. (2018). Targeting the PD-1/PD-L1 axis for the treatment of non-small-cell lung cancer. Curr. Oncol..

[56] Thorn M., Guha P., Cunetta M., Espat N.J., Miller G., Junghans R.P., Katz S.C. (2016). Tumor-associated GM-CSF overexpression induces immunoinhibitory molecules via STAT3 in myeloid-suppressor cells infiltrating liver metastases. Cancer Gene Ther..

[57] Buchbinder E.I., Desai A. (2016). CTLA-4 and PD-1 Pathways: Similarities, Differences, and Implications of Their Inhibition. Am. J. Clin. Oncol..

[58] Rotte A. (2019). Combination of CTLA-4 and PD-1 blockers for treatment of cancer. J. Exp. Clin. Cancer Res..

[59] Postow M.A., Chesney J., Pavlick A.C., Robert C., Grossmann K., McDermott D., Linette G.P., Meyer N., Giguere J.K., Agarwala S.S. (2015). Nivolumab and Ipilimumab versus Ipilimumab in Untreated Melanoma. N. Engl. J. Med..

[60] Hodi F.S., Chesney J., Pavlick A.C., Robert C., Grossmann K.F., McDermott D.F., Linette G.P., Meyer N., Giguere J.K., Agarwala S.S. (2016). Combined nivolumab and ipilimumab versus ipilimumab alone in patients with advanced melanoma: 2-year overall survival outcomes in a multicentre, randomised, controlled, phase 2 trial. Lancet Oncol..

[61] Larkin J., Chiarion-Sileni V., Gonzalez R., Grob J.J., Cowey C.L., Lao C.D., Schadendorf D., Dummer R., Smylie M., Rutkowski P. (2015). Combined Nivolumab and Ipilimumab or Monotherapy in Untreated Melanoma. N. Engl. J. Med..

[62] Wolchok J.D., Chiarion-Sileni V., Gonzalez R., Rutkowski P., Grob J.-J., Cowey C.L., Lao C.D., Wagstaff J., Schadendorf D., Ferrucci P.F. (2017). Overall Survival with Combined Nivolumab and Ipilimumab in Advanced Melanoma. N. Engl. J. Med..

[63] Hodi F.S., Chiarion-Sileni V., Gonzalez R., Grob J.-J., Rutkowski P., Cowey C.L., Lao C.D., Schadendorf D., Wagstaff J., Dummer R. (2018). Nivolumab plus ipilimumab or nivolumab alone versus ipilimumab alone in advanced melanoma (CheckMate 067): 4-year outcomes of a multicentre, randomised, phase 3 trial. Lancet Oncol..

[64] Johnson D.B., Chandra S., Sosman J.A. (2018). Immune Checkpoint Inhibitor Toxicity in 2018. JAMA.

[65] Duffy M.J., Crown J. (2019). Biomarkers for Predicting Response to Immunotherapy with Immune Checkpoint Inhibitors in Cancer Patients. Clin. Chem..

[66] Uboha N.V., Milhem M.M., Kovacs C., Amin A., Magley A., Das Purkayastha D., Piha-Paul S.A. (2019). Phase II study of spartalizumab (PDR001) and LAG525 in advanced solid tumors and hematologic malignancies. J. Clin. Oncol..

[67] Lakhani N., Spreafico A., Tolcher A., Rodon J., Janku F., Chandana S., Oliva M., Sharma M., Abdul-Karim R., Hansen U. (2020). 1019O Phase I studies of Sym021, an anti-PD-1 antibody, alone and in combination with Sym022 (anti-LAG-3) or Sym023 (anti-TIM-3). Ann. Oncol..

[68] Labrijn A.F., Janmaat M.L., Reichert J.M., Parren P.W.H.I. (2019). Bispecific antibodies: A mechanistic review of the pipeline. Nat. Rev. Drug Discov..

[69] Brischwein K., Parr L., Pflanz S., Volkland J., Lumsden J., Klinger M., Locher M., Hammond S.A., Kiener P., Kufer P. (2007). Strictly Target Cell-dependent Activation of T Cells by Bispecific Single-chain Antibody Constructs of the BiTE Class. J. Immunother..

[70] Brischwein K., Schlereth B., Guller B., Steiger C., Wolf A., Lutterbuese R., Offner S., Locher M., Urbig T., Raum T. (2006). MT110: A novel bispecific single-chain antibody construct with high efficacy in eradicating established tumors. Mol. Immunol..

[71] Lutterbuese R., Raum T., Kischel R., Hoffmann P., Mangold S., Rattel B., Friedrich M., Thomas O., Lorenczewski G., Rau D. (2010). T cell-engaging BiTE antibodies specific for EGFR potently eliminate KRAS- and BRAF-mutated colorectal cancer cells. Proc. Natl. Acad. Sci. USA.

[72] Torisu-Itakura H., Schoellhammer H.F., Sim M.-S., Irie R.F., Hausmann S., Raum T., Baeuerle P.A., Morton D.L. (2011). Redirected Lysis of Human Melanoma Cells by a MCSP/CD3-bispecific BiTE Antibody That Engages Patient-derived T Cells. J. Immunother..

[73] Klinger M., Brandl C., Zugmaier G., Hijazi Y., Bargou R.C., Topp M.S., Gökbuget N., Neumann S., Goebeler M., Viardot A. (2012). Immunopharmacologic response of patients with B-lineage acute lymphoblastic leukemia to continuous infusion of T cell-engaging CD19/CD3-bispecific BiTE antibody blinatumomab. Blood.

[74] Goebeler M.-E., Knop S., Viardot A., Kufer P., Topp M.S., Einsele H., Noppeney R., Hess G., Kallert S., Mackensen A. (2016). Bispecific T-Cell Engager (BiTE) Antibody Construct Blinatumomab for the Treatment of Patients with Relapsed/Refractory Non-Hodgkin Lymphoma: Final Results From a Phase I Study. J. Clin. Oncol..

[75] Donnelly O., Harrington K., Melcher A., Pandha H. (2013). Live viruses to treat cancer. J. R. Soc. Med..

[76] Kelly E., Russell S.J. (2007). History of Oncolytic Viruses: Genesis to Genetic Engineering. Mol. Ther..

[77] Zou Y., Luo Y., Zhang J., Xia N., Tan G., Huang C. (2019). Bibliometric analysis of oncolytic virus research, 2000 to 2018. Medicine.

[78] Fukuhara H., Ino Y., Todo T. (2016). Oncolytic virus therapy: A new era of cancer treatment at dawn. Off. J. Jpn. Cancer Assoc..

[79] Bommareddy P.K., Patel A., Hossain S., Kaufman H.L. (2016). Talimogene Laherparepvec (T-VEC) and Other Oncolytic Viruses for the Treatment of Melanoma. Am. J. Clin. Dermatol..

[80] Pylayeva-Gupta Y., Lee K.E., Hajdu C.H., Miller G., Bar-Sagi D. (2012). Oncogenic Kras-Induced GM-CSF Production Promotes the Development of Pancreatic Neoplasia. Cancer Cell.

[81] Jou J., Harrington K.J., Zocca M.-B., Ehrnrooth E., Cohen E.E. (2020). The Changing Landscape of Therapeutic Cancer Vaccines—Novel Platforms and Neoantigen Identification. Clin. Cancer Res..

[82] Lollini P.L., Cavallo F., Nanni P., Forni G. (2006). Vaccines for tumour prevention. Nat. Cancer.

[83] Morse M.A., Gwin W.R., Mitchell D.A. (2021). Vaccine Therapies for Cancer: Then and Now. Target. Oncol..

[84] DeMaria P.J., Bilusic M. (2019). Cancer Vaccines. Hematol. Oncol. Clin. N. Am..

[85] Lamm D.L., Blumenstein B.A., Crawford E.D., Montie J.E., Scardino P., Grossman H.B., Stanisic T.H., Smith J.A., Sullivan J., Sarosdy M.F. (1991). A Randomized Trial of Intravesical Doxorubicin and Immunotherapy with Bacille Calmette–Guérin for Transitional-Cell Carcinoma of the Bladder. N. Engl. J. Med..

[86] Laheru D., Lutz E., Burke J., Biedrzycki B., Solt S., Onners B., Tartakovsky I., Nemunaitis J., Le D., Sugar E. (2008). Allogeneic Granulocyte Macrophage Colony-Stimulating Factor–Secreting Tumor Immunotherapy Alone or in Sequence with Cyclophosphamide for Metastatic Pancreatic Cancer: A Pilot Study of Safety, Feasibility, and Immune Activation. Clin. Cancer Res..

[87] Lipson E.J., Sharfman W.H., Chen S., McMiller T.L., Pritchard T.S., Salas J.T., Sartorius-Mergenthaler S., Freed I., Ravi S., Wang H. (2015). Safety and immunologic correlates of Melanoma GVAX, a GM-CSF secreting allogeneic melanoma cell vaccine administered in the adjuvant setting. J. Transl. Med..

[88] Salgia R., Lynch T., Skarin A., Lucca J., Lynch C., Jung K., Hodi F.S., Jaklitsch M., Mentzer S., Swanson S. (2003). Vaccination With Irradiated Autologous Tumor Cells Engineered to Secrete Granulocyte-Macrophage Colony-Stimulating Factor Augments Antitumor Immunity in Some Patients With Metastatic Non–Small-Cell Lung Carcinoma. J. Clin. Oncol..

[89] Small E.J., Sacks N., Nemunaitis J., Urba W.J., Dula E., Centeno A.S., Nelson W.G., Ando D., Howard C., Borellini F. (2007). Granulocyte Macrophage Colony-Stimulating Factor–Secreting Allogeneic Cellular Immunotherapy for Hormone-Refractory Prostate Cancer. Clin. Cancer Res..

[90] Rosenberg S.A., Restifo N.P., Yang J.C., Morgan R.A., Dudley M.E. (2008). Adoptive cell transfer: A clinical path to effective cancer immunotherapy. Nat. Cancer.

[91] Dudley M.E., Rosenberg S.A. (2003). Adoptive-cell-transfer therapy for the treatment of patients with cancer. Nat. Rev. Cancer.

[92] Itzhaki O., Hovav E., Ziporen Y., Levy D., Kubi A., Zikich D., Hershkovitz L., Treves A.J., Shalmon B., Zippel D. (2011). Establishment and Large-scale Expansion of Minimally cultured “Young” Tumor Infiltrating Lymphocytes for Adoptive Transfer Therapy. J. Immunother..

[93] Muranski P., Boni A., Wrzesinski C., Citrin D., Rosenberg S.A., Childs R.W., Restifo N.P. (2006). Increased intensity lymphodepletion and adoptive immunotherapy—How far can we go?. Nat. Clin. Pract. Oncol..

[94] Rosenberg S.A., Packard B.S., Aebersold P.M., Solomon D., Topalian S.L., Toy S.T., Simon P., Lotze M.T., Yang J.C., Seipp C.A. (1988). Use of Tumor-Infiltrating Lymphocytes and Interleukin-2 in the Immunotherapy of Patients with Metastatic Melanoma: A preliminary report. N. Engl. J. Med..

[95] Besser M.J., Shapira-Frommer R., Itzhaki O., Treves A.J., Zippel D.B., Levy D., Kubi A., Shoshani N., Zikich D., Ohayon Y. (2013). Adoptive Transfer of Tumor-Infiltrating Lymphocytes in Patients with Metastatic Melanoma: Intent-to-Treat Analysis and Efficacy after Failure to Prior Immunotherapies. Clin. Cancer Res..

[96] Porter D.L., Levine B.L., Kalos M., Bagg A., June C.H. (2011). Chimeric Antigen Receptor–Modified T Cells in Chronic Lymphoid Leukemia. N. Engl. J. Med..

[97] Hartmann J., Schüßler-Lenz M., Bondanza A., Buchholz C.J. (2017). Clinical development of CAR T cells—Challenges and opportunities in translating innovative treatment concepts. EMBO Mol. Med..

[98] Chmielewski M., Abken H. (2015). TRUCKs: The fourth generation of CARs. Exp. Opin. Biol. Ther..

[99] Gargett T., Brown M.P. (2014). The inducible caspase-9 suicide gene system as a “safety switch” to limit on-target, off-tumor toxicities of chimeric antigen receptor T cells. Front. Pharmacol..

[100] Lorenzo-Herrero S., López-Soto A., Sordo-Bahamonde C., Gonzalez-Rodriguez S., Vitale M., Gonzalez S. (2018). NK Cell-Based Immunotherapy in Cancer Metastasis. Cancers.

[101] Hermanson D.L., Kaufman D.S. (2015). Utilizing Chimeric Antigen Receptors to Direct Natural Killer Cell Activity. Front. Immunol..

[102] Miller J.S., Soignier Y., Panoskaltsis-Mortari A., McNearney S.A., Yun G.H., Fautsch S.K., McKenna D., Le C., DeFor T.E., Burns L.J. (2005). Successful adoptive transfer and in vivo expansion of human haploidentical NK cells in patients with cancer. Blood.

[103] Geller M.A., Cooley S., Judson P.L., Ghebre R., Carson L.F., Argenta P.A., Jonson A.L., Panoskaltsis-Mortari A., Curtsinger J., McKenna D. (2011). A phase II study of allogeneic natural killer cell therapy to treat patients with recurrent ovarian and breast cancer. Cytotherapy.

[104] Veluchamy J.P., Kok N., van der Vliet H.J., Verheul H.M.W., De Gruijl T.D., Spanholtz J. (2017). The Rise of Allogeneic Natural Killer Cells as a Platform for Cancer Immunotherapy: Recent Innovations and Future Developments. Front. Immunol..

[105] Cichocki F., Bjordahl R., Gaidarova S., Mahmood S., Abujarour R., Wang H., Tuininga K., Felices M., Davis Z.B., Bendzick L. (2020). iPSC-derived NK cells maintain high cytotoxicity and enhance in vivo tumor control in concert with T cells and anti-PD-1 therapy. Sci. Transl. Med..

[106] Li Y., Hermanson D.L., Moriarity B.S., Kaufman D.S. (2018). Human iPSC-Derived Natural Killer Cells Engineered with Chimeric Antigen Receptors Enhance Anti-tumor Activity. Cell Stem Cell.

[107] Wilhelm M., Smetak M., Schaefer-Eckart K., Kimmel B., Birkmann J., Einsele H., Kunzmann V. (2014). Successful adoptive transfer and in vivo expansion of haploidentical gammadelta T cells. J. Transl. Med..

[108] Khairallah C., Chu T.H., Sheridan B.S. (2018). Tissue Adaptations of Memory and Tissue-Resident Gamma Delta T Cells. Front. Immunol..

[109] Deniger D.C., Switzer K., Mi T., Maiti S., Hurton L., Singh H., Huls H., Olivares S., Lee D.A., Champlin R.E. (2013). Bispecific T-cells expressing polyclonal repertoire of endogenous gammadelta T-cell receptors and introduced CD19-specific chimeric antigen receptor. Mol. Ther..

[110] Capsomidis A., Benthall G., Van Acker H.H., Fisher J., Kramer A.M., Abeln Z., Majani Y., Gileadi T., Wallace R., Gustafsson K. (2018). Chimeric Antigen Receptor-Engineered Human Gamma Delta T Cells: Enhanced Cytotoxicity with Retention of Cross Presentation. Mol. Ther..

[111] Lamers C.H., Klaver Y., Gratama J.W., Sleijfer S., Debets R. (2016). Treatment of metastatic renal cell carcinoma (mRCC) with CAIX CAR-engineered T-cells-a completed study overview. Biochem. Soc. Trans..

[112] Lamers C.H., Sleijfer S., van Steenbergen S., van Elzakker P., van Krimpen B., Groot C., Vulto A., Bakker M.D., Oosterwijk E., Debets R. (2013). Treatment of Metastatic Renal Cell Carcinoma With CAIX CAR-engineered T cells: Clinical Evaluation and Management of On-target Toxicity. Mol. Ther..

[113] Vierboom M.P., Bos G.M., Ooms M., Offringa R., Melief C.J. (2000). Cyclophosphamide enhances anti-tumor effect of wild-type p53-specific CTL. Int. J. Cancer.

[114] Reits E.A., Hodge J.W., Herberts C.A., Groothuis T.A., Chakraborty M., Wansley E.K., Camphausen K., Luiten R.M., De Ru A.H., Neijssen J. (2006). Radiation modulates the peptide repertoire, enhances MHC class I expression, and induces successful antitumor immunotherapy. J. Exp. Med..

[115] Ganss R., Ryschich E., Klar E., Arnold B., Hämmerling G.J. (2002). Combination of T-cell therapy and trigger of inflammation induces remodeling of the vasculature and tumor eradication. Cancer Res..

[116] Deng L., Liang H., Xu M., Yang X., Burnette B., Arina A., Li X.-D., Mauceri H., Beckett M., Darga T. (2014). STING-Dependent Cytosolic DNA Sensing Promotes Radiation-Induced Type I Interferon-Dependent Antitumor Immunity in Immunogenic Tumors. Immunity.

[117] Domschke C., Schneeweiss A., Stefanovic S., Wallwiener M., Heil J., Rom J., Sohn C., Beckhove P., Schuetz F. (2016). Cellular Immune Responses and Immune Escape Mechanisms in Breast Cancer: Determinants of Immunotherapy. Breast Care.

[118] Neagu M.R., Reardon D.A. (2015). An Update on the Role of Immunotherapy and Vaccine Strategies for Primary Brain Tumors. Curr. Treat. Options Oncol..

[119] Ajina A., Maher J. (2017). Prospects for combined use of oncolytic viruses and CAR T-cells. J. Immunother. Cancer.

[120] Nishio N., Diaconu I., Liu H., Cerullo V., Caruana I., Hoyos V., Bouchier-Hayes L., Savoldo B., Dotti G. (2014). Armed Oncolytic Virus Enhances Immune Functions of Chimeric Antigen Receptor–Modified T Cells in Solid Tumors. Cancer Res..

[121] Bonifant C., Jackson H.J., Brentjens R.J., Curran K.J. (2016). Toxicity and management in CAR T-cell therapy. Mol. Ther. Oncolytics.

[122] Curran K.J., Pegram H.J., Brentjens R.J. (2012). Chimeric antigen receptors for T cell immunotherapy: Current understanding and future directions. J. Gene Med..

[123] Lamers C.H., Sleijfer S., Vulto A.G., Kruit W.H., Kliffen M., Debets R., Gratama J.W., Stoter G., Oosterwijk E. (2006). Treatment of Metastatic Renal Cell Carcinoma With Autologous T-Lymphocytes Genetically Retargeted Against Carbonic Anhydrase IX: First Clinical Experience. J. Clin. Oncol..

[124] Parkhurst M.R., Yang J.C., Langan R.C., Dudley M.E., Nathan D.-A.N., Feldman S.A., Davis J.L., Morgan R.A., Merino M.J., Sherry R.M. (2011). T Cells Targeting Carcinoembryonic Antigen Can Mediate Regression of Metastatic Colorectal Cancer but Induce Severe Transient Colitis. Mol. Ther..

[125] Kershaw M.H., Westwood J.A., Parker L.L., Wang G., Eshhar Z., Mavroukakis S.A., White D.E., Wunderlich J.R., Canevari S., Rogers-Freezer L. (2006). A Phase I Study on Adoptive Immunotherapy Using Gene-Modified T Cells for Ovarian Cancer. Clin. Cancer Res..

